# Immune Determinants of MASLD Progression: From Immunometabolic Reprogramming to Fibrotic Transformation

**DOI:** 10.3390/biology15020148

**Published:** 2026-01-14

**Authors:** Senping Xu, Zhaoshan Zhang, Zhongquan Zhou, Jiawei Guo

**Affiliations:** 1 Department of Cardiology, The First Affiliated Hospital of Yangtze University, Jingzhou 434000, China; 2 Department of Pharmacology, School of Medicine, Yangtze University, Jingzhou 434023, China; 3 Department of Pharmacology, Cardiac and Cerebral Vascular Research Center, Zhongshan School of Medicine, Sun Yat-Sen University, Guangzhou 510080, China

**Keywords:** metabolic dysfunction-associated steatotic liver disease, adaptive immunity, fibrotic remodeling, immunometabolic reprogramming

## Abstract

Metabolic dysfunction-associated steatotic liver disease (MASLD) represents a progressive spectrum of metabolic liver injury in which immune activation, metabolic stress, and stromal remodeling evolve in a tightly interdependent manner. Although early disease stages are dominated by metabolic overload, accumulating evidence indicates that immunometabolic rewiring and fibro-inflammatory amplification critically shape the transition toward metabolic dysfunction-associated steatohepatitis (MASH) and advanced fibrosis. This review synthesizes emerging insights into how hepatocyte stress responses, innate and adaptive immune circuits, and extracellular matrix-producing stromal populations interact to form a dynamic, feed-forward network driving disease progression. Particular emphasis is placed on the deterministic role of immune–fibrotic coupling in shaping clinical phenotypes, disease trajectory, and therapeutic responsiveness. Rather than focusing on individual molecular layers, we highlight how integrated clinical, imaging, and biomarker-informed frameworks can capture immune–fibrotic signatures relevant to risk stratification and precision intervention. Building on this systems-level perspective, we outline next-generation therapeutic strategies targeting immunometabolic circuits, cross-organ communication, and multi-system dysfunction. Finally, we discuss how future precision medicine—supported by integrative biomarker profiling and dynamic physiological assessment—may reshape MASLD management and improve long-term hepatic and cardiometabolic outcomes.

## Simple Summary

Metabolic dysfunction-associated steatotic liver disease (MASLD) can progress from simple steatosis to inflammatory injury and fibrosis. Beyond lipid accumulation, immune activation and fibrotic remodeling have emerged as key determinants of disease progression. This review synthesizes recent evidence showing how metabolic stress reshapes immune responses and stromal cell interactions, forming immune–fibrotic networks that drive disease evolution. Emphasis is placed on integrated clinical, imaging, and biomarker-based frameworks to capture immune–fibrotic signatures relevant to disease stratification and therapeutic response.

## 1. Introduction

Metabolic dysfunction-associated steatotic liver disease (MASLD) has become the most prevalent chronic liver disorder worldwide, paralleling the rapid growth of obesity, insulin resistance, and cardiometabolic syndromes [1]. Once considered a largely hepatic manifestation of metabolic stress, MASLD is now recognized as a systemic disease involving coordinated disturbances across metabolic, immune, and stromal compartments [2]. The evolution from simple steatosis to MASH and progressive fibrosis is not governed by lipid accumulation alone but rather by a complex interplay of hepatocellular injury, immunometabolic activation, and microenvironmental remodeling [3]. These processes collectively define the inflammatory tone of the liver and shape the fibrotic trajectory that determines long-term clinical outcomes.

Recent advances have uncovered previously unappreciated dimensions of MASLD pathobiology, including lineage-specific immune cell reprogramming, metabolic checkpoint–driven activation states, spatially segregated fibro-inflammatory niches, and multi-organ communication involving the gut, adipose tissue, cardiovascular system, kidneys, and the neuro-endocrine axis [4]. The convergence of these pathways forms an immunometabolic scaffold, defined here as the integrated metabolic, immune, and stromal microenvironment shaped by hepatocyte lipid stress, inflammatory signaling, and extracellular matrix remodeling, which collectively dictates fibrotic responsiveness in the liver [5]. In MASLD, this scaffold can be evaluated through quantifiable parameters such as hepatic fat burden (MRI-PDFF), inflammatory mediators (e.g., TNF-α, IL-1β), metabolic indices, and fibrosis-related readouts, and accelerates the transition from metabolic dysfunction to clinically significant disease [6]. This expanded conceptual framework provides opportunities to reassess disease staging, identify mechanistically anchored biomarkers, and design precision therapies that target the drivers of fibrotic progression rather than its downstream clinical manifestations [7].

In this review, we integrate recent insights into immunometabolic stress responses, inflammatory amplification loops, and fibro-immune crosstalk that govern MASLD progression. We highlight how immune–fibrotic coupling, referring to coordinated interactions between hepatic immune cells (e.g., macrophages) and fibrogenic stromal compartments, emerges as a deterministic driver, defined as a dominant and reproducible pathogenic influence that shapes disease severity, progression rate, and therapeutic responsiveness within specific MASLD subgroups. Based on these mechanistic foundations, we discuss innovative strategies aimed at rewiring immunometabolic circuits and coordinating multi-organ interventions to achieve genuine precision therapy for MASLD. This systems-level perspective reflects a shift toward understanding MASLD not as an isolated hepatic condition but as a dynamic, multi-system disease requiring equally integrative therapeutic solutions.

## 2. Immunometabolic Checkpoints Governing MASLD Initiation

### 2.1. Hepatic Metabolic Stress as the Immune Priming Signal

**Figure 1:**
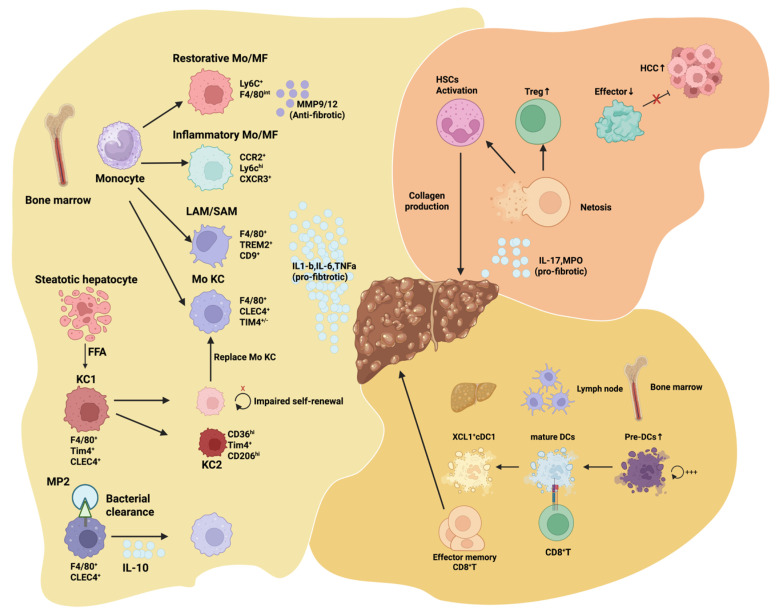
Key Myeloid Cell-Mediated Mechanisms Driving Immune and Structural Remodeling in NASLD.

Metabolic stress within the liver constitutes the earliest and most decisive trigger for immune activation in MASLD [8]. As hepatocytes experience lipid overload and mitochondrial dysfunction, the accumulation of enlarged lipid droplets generates oxidative stress, leading to excessive reactive oxygen species (ROS) formation, mitochondrial DNA leakage, and the release of multiple damage-associated molecular patterns (DAMPs) (Figure 1) [9]. These endogenous danger signals stimulate the hepatic innate immune sensing network and induce an early state of alertness in both resident and infiltrating immune cells, long before clinically overt inflammation becomes apparent (Figure 1).

Among these early responders, macrophages are the first to detect metabolic disturbances (Figure 1). Recognition of ROS and DAMPs through pattern recognition receptors enhances their inflammatory gene expression, reshapes their metabolic programs, and primes them for amplified downstream responses [10]. Neutrophils also exhibit heightened sensitivity to metabolic danger cues; their recruitment threshold decreases under metabolic stress, allowing them to infiltrate the liver during minor injury stages and release proteases and neutrophil extracellular traps (NETs), thereby modifying the local inflammatory milieu9 (Figure 1) [11]. Meanwhile, dendritic cells increase antigen uptake and presentation capacity under metabolic stress and shift toward a proinflammatory secretory profile, establishing the foundation for subsequent activation of adaptive immunity (Figure 1) [12].

Thus, metabolic stress functions not merely as a background condition but as a key signal that determines the immunological threshold [13]. Danger signals originating from hepatocytes recalibrate the activation sensitivity of macrophages, neutrophils, and dendritic cells, effectively priming the immune system toward a proinflammatory trajectory rather than maintaining tolerogenic homeostasis [14]. This early phase of “immune pre-activation” dictates whether inflammation will escalate, persist, and ultimately drive MASLD toward the more severe and destructive phase of MASH.

A comprehensive understanding of how metabolic stress programs this innate immune activation threshold is therefore essential for identifying disease inflection points and developing effective, preventive therapeutic strategies. Together, these observations position hepatocyte metabolic stress as the primary upstream signal, necessitating specialized immune sentinels to interpret and propagate this disturbance within the liver microenvironment.

This schematic summarizes the central innate immune regulatory pathways mediated by macrophages, neutrophils (Neu), and dendritic cells (DCs) during the development and progression of MASLD. Hepatocytes under metabolic stress and lipotoxicity release a spectrum of DAMPs, together with free fatty acids, oxidative stress products, and mitochondrial components, thereby activating hepatic myeloid populations. Recruited monocytes differentiate into multiple macrophage subsets within steatotic and fibrotic microenvironments, where pro-inflammatory and pro-fibrotic phenotypes amplify local cytokine and chemokine networks, modulate hepatic stellate cell (HSC) activation, drive extracellular matrix (ECM) deposition, and promote the formation of organized fibrotic foci. Neutrophils infiltrate the liver early in MASLD and, through the release of proteases, reactive oxygen species, and the formation of neutrophil extracellular traps (NETs), exacerbate hepatocellular stress and accelerate the fibrogenic conversion of HSCs. Persistent NET formation sustains chronic inflammatory amplification loops and contributes to structural tissue remodeling. Concurrently, dendritic cells undergo maturation and functional reprogramming in response to metabolic and inflammatory cues, shaping cytokine networks that influence monocyte-to-macrophage differentiation trajectories and determine the recruitment and activation intensity of other innate immune cells. Interactions between DCs and stromal components, including liver sinusoidal endothelial cells and HSCs, modulate the balance between inflammation and repair, thereby steering the progression from steatosis to steatohepatitis and fibrosis. Collectively, these myeloid populations integrate metabolic danger signals and innate immune activation to form a core regulatory axis that drives inflammatory propagation and structural remodeling in MASLD.

### 2.2. Metabolic Stress-Induced Kupffer Cell Reprogramming and Immune Activation

**Figure 2:**
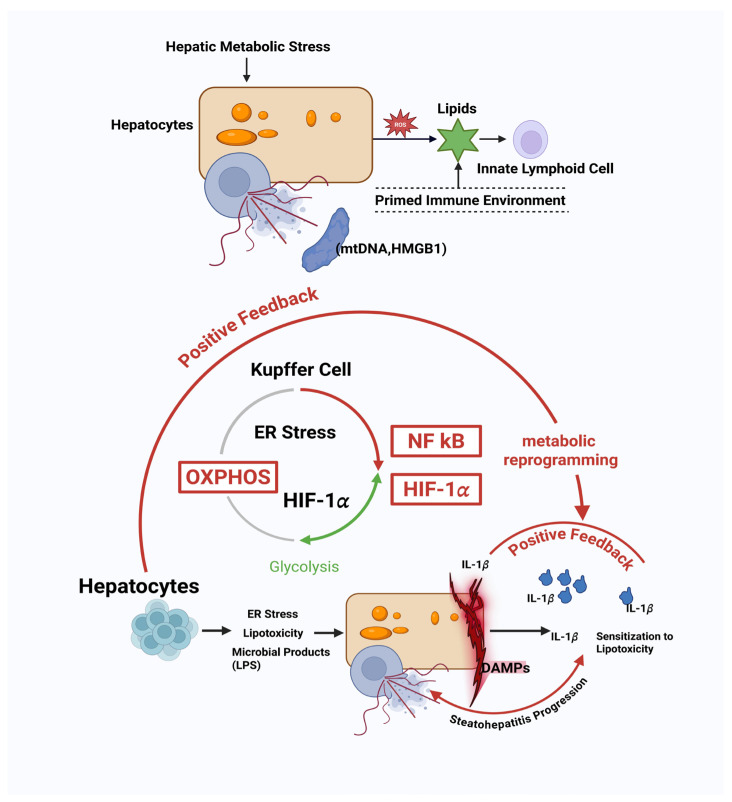
MASLD: Hepatic Immune-Metabolic Checkpoints Governing Initiation and Progression.

Kupffer cells (KCs), as the resident macrophages of the liver, are central regulators of immune surveillance and homeostasis [15]. In the context of MASLD, hepatic lipid overload and oxidative stress do not merely trigger an inflammatory response; they actively reprogram the metabolic state of KCs, which in turn dictates their functional polarization and cytokine output (Figure 2) [16]. Specifically, excessive free fatty acids and cholesterol derivatives induce a metabolic shift in KCs from oxidative phosphorylation (OXPHOS) toward glycolysis [17,18]. This shift is accompanied by accumulation of lipid intermediates such as ceramides and diacylglycerols, which further modulate signaling pathways including NF-κB and HIF-1α [19].

Metabolic reprogramming of KCs does not simply correspond to a classical M1/M2 polarization [20]. Rather, it establishes a context-dependent immune tone, characterized by a spectrum of states ranging from “lipid-tolerant” to “lipid-sensitized” phenotypes [21]. Lipid-tolerant KCs maintain relative immune quiescence despite metabolic stress, whereas lipid-sensitized KCs exhibit heightened responsiveness to DAMPs and cytokines, amplifying early inflammatory signals (Figure 2) [22]. This functional heterogeneity among KCs determines whether the liver microenvironment remains stable or shifts toward a pro-inflammatory milieu, setting the stage for subsequent recruitment of monocyte-derived macrophages and lymphocytes [23].

Moreover, KC metabolic reprogramming interacts with hepatocyte-derived signals, forming a feed-forward loop that amplifies immune activation [24]. For example, ceramide accumulation in KCs can induce IL-1β secretion, which not only reinforces KC activation but also sensitizes neighboring hepatocytes to lipotoxic stress, thereby perpetuating DAMP release [25]. These early events collectively prime the hepatic immune landscape, defining the trajectory toward either resolution or progression to steatohepatitis.

In summary, Kupffer cell metabolic reprogramming is a critical checkpoint in MASLD initiation, translating metabolic perturbations into immune system decisions and determining the early tone of liver immunity [23]. Targeting this reprogramming may offer a promising strategy for early intervention before irreversible liver damage occurs [26]. While Kupffer cell reprogramming constitutes a central intrahepatic checkpoint, immune activation in MASLD is further shaped by extrahepatic inputs that modulate innate sensing thresholds and inflammatory tone [27].

### 2.3. Gut–Liver Axis and Immune Remodeling

Lipotoxic hepatocytes actively participate in the initiation and amplification of immune responses in MASLD, establishing a complex crosstalk with innate immune sensors that drives early disease progression (Figure 2) [28]. Excessive accumulation of free fatty acids, cholesterol crystals, and toxic lipid intermediates induces hepatocyte stress responses, including endoplasmic reticulum (ER) stress, mitochondrial dysfunction, and oxidative damage [29]. These stress responses lead to the release of a diverse array of DAMPs such as mitochondrial DNA, oxidized phospholipids, ATP, and high-mobility group box 1 (HMGB1), as well as pro-inflammatory extracellular vesicles [30].

These hepatocyte-derived signals engage pattern recognition receptors (PRRs) on resident innate immune cells, including Toll-like receptors (TLRs), NOD-like receptors (NLRs), and C-type lectin receptors (CLRs) (Figure 2) [31]. Activation of these receptors triggers multiple downstream pathways, such as NF-κB, MAPK, and NLRP3 inflammasome signaling, which orchestrate the initial production of cytokines and chemokines [32]. Notably, the type, magnitude, and temporal pattern of hepatocyte-derived signals determine the qualitative nature of the immune response [33]. Moderate stress may induce tolerogenic signals that maintain hepatic homeostasis, whereas sustained or excessive lipotoxic stress shifts the immune landscape toward a pro-inflammatory state, increasing susceptibility to further hepatocyte injury [34].

Emerging evidence indicates that the gut-liver axis plays a critical role in shaping hepatic immune responses in MASLD [35]. Gut-derived microbial metabolites, including short-chain fatty acids (SCFAs), bile acids, and tryptophan derivatives, can modulate both resident and circulating immune cells [36]. SCFAs, produced by bacterial fermentation of dietary fibers, promote regulatory macrophage and dendritic cell phenotypes, thereby supporting hepatic immune tolerance [37]. Bile acids, through activation of farnesoid X receptor (FXR) and Takeda G-protein receptor 5 (TGR5), influence Kupffer cell polarization and cytokine production, linking nutrient and microbial sensing to immune activation [38]. Tryptophan metabolites, such as indole derivatives, act on the aryl hydrocarbon receptor (AhR) in innate immune cells, fine-tuning inflammatory responses and maintaining hepatic homeostasis [39]. Collectively, these microbial signals interact with hepatocyte-derived danger signals to orchestrate a multi-layered immune remodeling process, integrating metabolic stress and microbial cues in early MASLD progression [40].

The crosstalk between hepatocytes and innate immune cells is further reinforced by a feed-forward loop. Activated Kupffer cells and dendritic cells produce IL-1β, TNF-α, and CCL2, which enhance hepatocyte stress responses and recruit circulating monocytes and neutrophils into the liver (Figure 2) [41]. This loop amplifies inflammation and contributes to the progression from simple steatosis to MASH [42]. Additionally, hepatocyte-derived extracellular vesicles carry lipids, microRNAs, and other regulatory molecules that can modulate the metabolic state, gene expression, and effector functions of innate immune cells, creating a bidirectional communication network [43].

Emerging evidence also suggests that subcellular localization and composition of lipotoxic signals influence the spatial organization of innate immune activation. For instance, pericentral hepatocytes often generate different DAMP profiles compared to periportal hepatocytes, which may explain the zonal heterogeneity of inflammation observed in early MASLD [44]. This spatially resolved hepatocyte-immune crosstalk establishes a structured immune microenvironment that dictates the trajectory of early disease progression and primes the liver for subsequent recruitment and activation of adaptive immune components [45].

In summary, hepatocyte-innate immune crosstalk is not a simple trigger-response mechanism; rather, it constitutes a finely tuned, multi-layered network in which the type, quantity, and context of lipotoxic signals determine immune cell activation, local cytokine production, and ultimately the trajectory of MASLD progression [46].

### 2.4. Innate Lymphoid Cells as Early Metabolic Interpreters

Innate lymphoid cells (ILCs) are a heterogeneous group of tissue-resident immune cells that play a crucial role in sensing environmental changes and orchestrating early immune responses [47]. In the context of MASLD, ILCs act as metabolic interpreters, rapidly translating hepatocyte-derived stress signals into immune outputs that shape the trajectory of disease progression [48]. Unlike adaptive lymphocytes, ILCs do not require antigen-specific priming, which allows them to respond promptly to lipotoxic stress and DAMPs released by hepatocytes [47].

The three major ILC subsets—ILC1, ILC2, and ILC3—display distinct metabolic dependencies and functional repertoires (Figure 2) [49]. ILC1s, which are traditionally cytotoxic, are influenced by intracellular lipid handling and mitochondrial ROS levels, modulating their IFN-γ production [50,51]. ILC2s, which are associated with tissue repair and type 2 immunity, sense fatty acid-derived metabolites and respond by producing IL-5 and IL-13, thereby contributing to early recruitment of eosinophils and modulation of macrophage phenotypes [52]. ILC3s detect microbial metabolites and bile acid derivatives via the aryl hydrocarbon receptor (AhR) and produce IL-17 and IL-22, which influence both hepatocyte survival and stromal remodeling [53,54].

ILC activation is highly sensitive to the quality, quantity, and temporal dynamics of metabolic cues. For instance, transient lipotoxic stress may induce a tolerogenic ILC2 response that favors tissue repair, whereas sustained lipid overload skews ILC2 and ILC3 activities toward pro-inflammatory and pro-fibrotic outputs (Figure 2) [55]. Moreover, ILCs communicate bidirectionally with Kupffer cells and dendritic cells, integrating signals from both hepatocytes and the local immune milieu to calibrate cytokine networks and orchestrate early immune landscapes [56].

Emerging studies suggest that ILCs also undergo metabolic reprogramming in response to local lipid and glucose availability. Such reprogramming affects their proliferation, survival, and effector functions, creating a feedback loop that links hepatocyte metabolic state to immune system behavior [57]. Therefore, ILCs function as early metabolic sensors and translators, bridging hepatocyte stress to systemic immune responses and shaping the initial trajectory of MASLD toward either resolution or progression (Figure 2) [58].

In summary, ILCs act as critical early interpreters of hepatocyte-derived metabolic stress, translating lipid and danger signals into tailored cytokine responses that set the stage for subsequent immune recruitment, inflammation, and eventual fibrosis [59]. Collectively, these parallel immune interpreters do not operate in isolation but converge to determine whether metabolic stress remains tolerable or escalates beyond a critical immune activation threshold.

### 2.5. MASLD as an Immune-Threshold Phenomenon

Emerging evidence suggests that the initiation and early progression of MASLD can be conceptualized as an immune-threshold phenomenon, in which the magnitude, duration, and quality of metabolic stress determine whether the hepatic immune system remains tolerant or transitions into a pro-inflammatory state [60]. Unlike a linear model where lipid accumulation gradually triggers inflammation, the immune-threshold model emphasizes a discrete tipping point: once hepatocyte-derived danger signals surpass a certain threshold, innate immune cells including Kupffer cells, dendritic cells, and ILCs collectively shift their functional state, creating a self-reinforcing pro-inflammatory microenvironment [61,62].

This threshold is influenced by multiple factors. First, the intensity of metabolic perturbation—such as the degree of lipotoxicity, oxidative stress, and cholesterol crystallization—determines the strength of DAMP release and PRR activation [63]. Second, immune cell metabolic programming sets the sensitivity of innate cells to these signals; for instance, lipid-sensitized Kupffer cells or metabolically reprogrammed ILCs respond more vigorously to subthreshold stress, effectively lowering the system’s immune threshold [47]. Third, spatial heterogeneity within the liver, including zonal differences in hepatocyte stress and local immune cell density, modulates how the immune threshold is reached and propagated [64].

Once the immune threshold is crossed, a feed-forward amplification loop emerges: innate immune activation promotes further hepatocyte stress, cytokine secretion, and recruitment of monocytes and neutrophils, rapidly escalating inflammation and predisposing the tissue toward MASH (Figure 2) [65]. Importantly, the immune-threshold concept explains why some individuals with moderate hepatic steatosis do not progress, whereas others with similar lipid levels rapidly develop MASH [66]. It underscores that disease progression is not solely determined by the quantity of fat accumulation, but by the integrated response of the immune system to metabolic stress [67].

In this framework, early interventions should aim to modulate immune sensitivity and recalibrate the hepatic immune threshold, rather than simply reducing lipid burden [68]. Strategies may include targeting metabolic checkpoints in Kupffer cells and ILCs, blocking key PRR signaling pathways, or enhancing tolerogenic signals that preserve immune homeostasis [69]. By understanding MASLD as an immune-threshold phenomenon, researchers and clinicians can identify critical early-stage checkpoints for preventing irreversible liver injury [70].

Hepatic metabolic stress constitutes the critical priming signal for MASLD initiation. Excessive lipid accumulation and mitochondrial dysfunction in hepatocytes elevate reactive oxygen species ROS, inducing the leakage of mitochondrial DNA (mtDNA) and the release of DAMPs, such as HMGB1. These signals prime the liver microenvironment, including ILCs, shifting it toward a pro-inflammatory state. Subsequently, KCs undergo metabolic reprogramming, switching from oxidative phosphorylation (OXPHOS) to glycolysis. This process is driven by accumulating lipid intermediates, activating pathways like NF-kB and HIF-1α. Functionally reprogrammed KCs secrete high levels of IL-1β, which, in conjunction with translocated microbial products (LPS) and ER stress in hepatocytes, forms a positive feedback loop to amplify inflammation. Collectively, this mechanism drives sensitization to lipotoxicity and the progression toward steatohepatitis, representing a key checkpoint for irreversible liver damage.

## 3. Adaptive Immunity Rewiring and Disease Transition to MASH

While Section 2 established the early innate immune and metabolic landscape of MASLD, Section 3 focuses on how these upstream signals shape adaptive immune reprogramming and drive the transition to MASH. During the transition from simple steatosis to inflammatory liver injury, innate immune activation is not the endpoint but rather the ignition switch for a deeper layer of immune reprogramming [71]. As summarized in Section 2, metabolic stress, lipotoxic cell damage, hepatocyte-derived DAMPs, and the activation of Kupffer cells, dendritic cells, and innate lymphoid cells collectively establish a highly pro-inflammatory hepatic microenvironment. This environment not only amplifies local inflammation but also provides the necessary priming signals for the activation, expansion, and phenotypic diversification of adaptive immune cells [72].

Once these innate immune signals surpass a critical threshold, T and B cells no longer maintain their homeostatic surveillance roles [73]. Instead, they undergo profound metabolic and functional reprogramming: T cells shift from regulatory to effector phenotypes, and B cells transition from tolerogenic states to antibody- and cytokine-driven pro-inflammatory programs [74]. This cascade—characterized by “immune stress, metabolic rewiring, and effector polarization”—becomes a decisive force driving the progression from NAFL to MASH [75]. Therefore, in Section 3, we focus on how adaptive immunity is reshaped within the lipotoxic and innate immunity–activated milieu, and how such rewiring accelerates inflammatory progression and fibrotic transformation in the liver.

### 3.1. Priming of Adaptive Immunity by Lipotoxic and Innate Immune Signals

As the lipotoxic microenvironment intensifies, the liver does not merely suffer inflammatory insults passively; instead, it gradually acquires the capacity to sculpt adaptive immune activation [76]. Pro-inflammatory mediators released by innate immune cells—such as IL-1β, TNF-α, and IL-6—together with hepatocyte-derived DAMPs, sensitize the hepatic antigen-presenting machinery [77]. Consequently, dendritic cells, Kupffer cells, and liver sinusoidal endothelial cells present a broader and more immunogenic spectrum of antigens, placing incoming T cells into a highly activatable and polarization-prone state from the moment they enter the hepatic niche [78].

Lipid and cholesterol accumulation further reprogram dendritic cells metabolically, driving them toward phenotypes characterized by elevated co-stimulatory molecule expression and enhanced effector T-cell priming capacity [79]. The antigens they present are no longer restricted to classical pathogen-derived components; instead, they increasingly include hepatocyte-derived elements generated under lipotoxic stress [80]. This shift promotes a quasi-autoimmune mode of T-cell activation, in which liver-specific antigens induce persistent, self-sustaining immune responses [81].

In parallel, gut dysbiosis introduces additional exogenous signals—such as LPS and bacterial metabolites—through the portal circulation, exposing adaptive immune cells to a composite antigenic stimulus [82]. This convergence of endogenous and exogenous antigen pressure gradually drives hepatic T and B cells out of homeostatic regulation and into a mode of amplified and persistent reactivity [83]. In this context, the initiation of adaptive immunity becomes a multifactorial process shaped jointly by lipotoxicity, cellular injury, microbial signals, and innate immune activation, laying the groundwork for subsequent effector polarization and the transition toward necroinflammation and fibrosis [84].

### 3.2. Metabolic Reprogramming of Adaptive Lymphocytes in the Lipotoxic Liver

Within the chronically lipotoxic hepatic environment, adaptive lymphocytes undergo profound metabolic reprogramming that reshapes their functional identity and ultimately dictates their contribution to disease progression [85]. Upon entering a milieu rich in free fatty acids, cholesterol crystals, and oxidized lipids, T cells experience mitochondrial dysfunction and oxidative stress that drive a shift from oxidative phosphorylation toward a highly glycolysis-dependent metabolic profile [86]. This glycolytic acceleration not only fuels rapid proliferation but also biases differentiation toward inflammatory effector phenotypes such as Th1 and Th17 cells, while regulatory T cells—more dependent on fatty acid oxidation—are selectively disadvantaged, leading to a progressive collapse of immunoregulatory balance [87]. In parallel, dysregulated cholesterol metabolism exposes T cells to oxidized LDL and lipid peroxidation products that alter membrane composition, amplify TCR signaling, and support sustained activation, forming a self-perpetuating inflammatory circuit [88].

B cells are similarly shaped by lipid overload; increased intracellular lipid droplets and cholesterol accumulation enhance their antigen-presenting capacity and pro-inflammatory cytokine production, promoting differentiation into plasma cells or inflammatory B-cell subsets [89]. At the same time, lipotoxic stress depletes regulatory B cells, weakening tolerogenic pathways and allowing antibody-dependent inflammatory processes to expand under chronic antigenic stimulation [90]. Importantly, metabolic reprogramming of T and B cells is not isolated but interwoven through cytokine crosstalk, antigen presentation, and local metabolic byproducts [91]. Effector T-cell–derived IFN-γ and IL-17 further worsen hepatocellular lipid accumulation and oxidative injury, amplifying lipotoxic signals that continually reinforce high metabolic and hyperreactive states in adaptive lymphocytes [92].

Collectively, these metabolic alterations transform adaptive immune cells from guardians of hepatic homeostasis into persistent, energetically empowered drivers of necroinflammation [93]. Through this tight coupling of metabolism and effector programming, adaptive immunity becomes a central force propelling the transition from steatosis to MASH [94].

### 3.3. B Cells as Drivers of Immune Amplification and Fibrotic Signaling

In MASLD progression, B cells not only perform classical antibody production but also serve as central regulators of the local immune microenvironment and fibrotic signaling [95]. Chronic lipotoxic stress and persistent low-level antigen stimulation drive B cell activation, resulting in upregulation of surface co-stimulatory molecules CD40 and CD80/CD86, while engaging BCR-mediated NF-κB, PI3K-Akt, and MAPK pathways to sustain proliferation and survival [96]. Activated B cells secrete cytokines such as TNF-α, IL-6, and lymphotoxin-α/β, which in turn activate macrophages and dendritic cells, promoting the production of TGF-β and PDGF and enhancing hepatic stellate cell (HSC) proliferation and ECM deposition [97]. Chemokines released by B cells, including CCL2 and CXCL13, facilitate the targeted recruitment of monocytes and lymphocytes to defined hepatic regions, creating high-density immune microdomains that amplify local intercellular signaling [98].

Furthermore, B cells can form tertiary lymphoid structure-like niches within the liver, providing spatial hubs for antigen presentation, cytokine amplification, and signal integration [99]. Within these microenvironments, B cells directly act on HSCs via LTβR/NF-κB signaling, inducing differentiation into activated myofibroblasts and upregulation of collagen I/III, fibronectin, and α-SMA, thereby driving ECM accumulation and fibrotic progression [100]. B cells also modulate HSC metabolic state through PI3K-Akt signaling, enhancing responsiveness to local pro-fibrotic cues [101]. Collectively, B cells integrate antigenic signals, cytokine networks, and stromal feedback to tightly couple adaptive immune activation with tissue remodeling, positioning them as pivotal mediators of MASLD progression and local fibrosis [102].

### 3.4. T Cells as Regulators of Inflammation-Fibrosis Crosstalk

**Figure 3:**
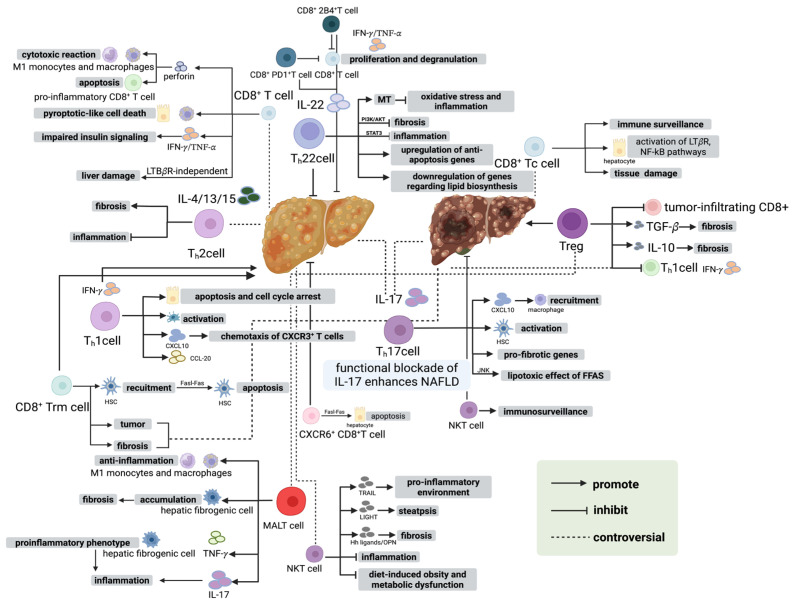
Integrated landscape of adaptive T-cell dysregulation driving MASH progression and transition to HCC.

T cells play a central role in the progression from MASLD to MASH, not only through direct cytotoxic effects on hepatocytes but also by orchestrating dynamic interactions between the immune microenvironment and hepatic stromal compartments [103]. Distinct effector T cell subsets secrete pro-inflammatory and regulatory cytokines, including IFN-γ, TNF-α, IL-17, and IL-22, and engage with hepatic stellate cells (HSCs), liver sinusoidal endothelial cells, and the ECM via surface receptors such as FasL, CCR5, and CXCR6 (Figure 3) [104]. These interactions activate intracellular signaling pathways in HSCs, including JAK-STAT, NF-κB, MAPK, and PI3K-Akt, promoting HSC proliferation, migration, and differentiation while upregulating fibrogenic markers such as collagen I/III, fibronectin, and α-SMA (Figure 3) [105,106]. Importantly, these mechanisms can drive fibrotic phenotypes even in the absence of overt hepatocyte apoptosis, linking adaptive immune activation to structural remodeling of the liver [107].

Moreover, T cells integrate local metabolic byproducts (e.g., fatty acids, acetate), oxidative stress signals, and chemokine gradients to regulate vascular permeability, ECM composition, and the redox microenvironment, thereby coordinating sustained immune cell recruitment and stromal activation [108]. Recruited macrophages and neutrophils further amplify this response by releasing IL-1β, IL-6, TGF-β, and PDGF, establishing a self-reinforcing positive feedback loop that enhances chronic inflammation and ECM deposition (Figure 3) [109]. T cells also modulate HSC metabolic reprogramming, including glucose utilization, lipid handling, and oxidative phosphorylation, contributing to spatial heterogeneity in fibrotic responses (Figure 3) [110]. Together, these mechanisms demonstrate that T cells function not merely as classical cytotoxic or helper cells, but as a central hub integrating immune and stromal signals, translating chronic immune activation into coordinated tissue remodeling and driving the progression and fibrosis characteristic of MASH [111].

This schematic summarizes the coordinated yet highly heterogeneous roles of CD4^+^ T cells, CD8^+^ T cells, and unconventional T cells in shaping the immunopathological continuum of NASLD. CD4^+^ T-cell subsets modulate hepatocellular injury, inflammation, fibrogenesis, and tumor surveillance through distinct cytokine programs and context-dependent interactions with macrophages, hepatic stellate cells, and hepatocytes. CD8^+^ T-cell populations exhibit dual damage-repair dynamics, with resident memory, cytotoxic, and dysregulated effector subsets variably contributing to hepatocyte death, metabolic impairment, fibrosis modulation, and oncogenic transformation. Unconventional T cells—including NKT, MAIT cells—integrate metabolic cues with innate-like effector mechanisms to either constrain or amplify steatosis, fibrotic remodeling, and inflammatory microdomains. Together, these T-cell networks form a multilayered regulatory axis that determines whether MASLD evolves toward controlled immune adaptation, progressive steatohepatitis, fibrosis, or MASH-associated hepatocellular carcinoma. Abbreviations: HSC, hepatic stellate cell; KCs, Kupffer cells; MAIT, mucosal-associated invariant T cell; NKT, natural killer T cell; Trm, tissue-resident memory T cell.

### 3.5. Loss of Immune Resolution and Establishment of Chronic Inflammatory Loops

In the progression to MASH, adaptive immunity not only becomes hyperactive but also loses its intrinsic capacity to resolve inflammation, establishing self-sustaining inflammatory loops that persist independently of initial triggers (Table 1) [112]. Regulatory networks that normally maintain tolerance, including Treg- and Breg-mediated checkpoints, undergo phenotypic destabilization: suppressive cytokine production declines, checkpoint receptor expression is altered, and epigenetic programs maintaining regulatory identity are disrupted [113]. This collapse of immunoregulatory mechanisms permits even minor antigenic or mechanical stress to elicit disproportionate immune responses (Table 1) [114]. Beyond cytokine-mediated effects, adaptive immune cells engage in continuous cross-talk with the remodeled extracellular matrix, activated stellate cells, and altered sinusoidal endothelium, which provides persistent co-stimulatory and adhesion signals that stabilize immune cell retention and activation [115]. Over time, these interactions establish feedback circuits in which immune cells continuously reinforce local tissue activation, ECM remodeling, and fibrogenic signaling, creating a chronic inflammatory milieu that is self-perpetuating (Table 1) [116]. Importantly, this process occurs without reliance on previously described metabolic or spatial mechanisms, highlighting a distinct layer of immune dysregulation that transforms adaptive immunity from a protective system into a driver of irreversible liver pathology [117].

## 4. Cytokine-Guided Stromal and Fibrotic Remodeling

Chronic dysregulation of adaptive immunity creates a persistent inflammatory environment that extends beyond classical effector functions. Effector T and B cells, together with impaired regulatory networks, produce a diverse array of cytokines and chemokines that act on hepatic stellate cells, endothelial cells, and the extracellular matrix [134]. These soluble mediators coordinate stromal cell activation, tissue remodeling, and fibrotic responses, translating sustained immune activity into structural alterations characteristic of progressive MASH [135]. This perspective highlights the central role of cytokine-mediated signaling in linking immune dysregulation to the initiation and propagation of fibrotic remodeling.

### 4.1. Cytokine Signatures Linking Immune Cells and Hepatic Stellate Cells

Beyond HSCs, liver sinusoidal endothelial cells and other mesenchymal/stromal populations serve as critical integrators of immune signals and modulators of fibrogenesis [136]. LSECs respond dynamically to cytokines (e.g., TNF-α, IL-1β, IFN-γ), chemokines, and hepatocyte-derived metabolic products, adjusting their expression of adhesion molecules, antigen-presenting capacity, and angiocrine factors [137]. These changes guide the spatial recruitment, retention, and localization of innate and adaptive immune cells, thereby creating specialized microdomains where immune-stromal interactions are concentrated [138].

Stellate cell heterogeneity further shapes fibrosis outcomes. Different HSC subsets exhibit variable receptor expression, metabolic profiles, and sensitivity to cytokines or growth factors, resulting in zone-specific ECM deposition, stiffness gradients, and differential fibrogenic capacity [139]. Portal fibroblasts, perivascular mesenchymal cells, and other non-immune stromal populations actively contribute to this process by secreting chemokines (CCL2, CXCL12), cytokines (TGF-β, PDGF), and matrix-modifying enzymes, reinforcing immune cell activation and establishing self-perpetuating fibrotic niches [140].

Spatial organization is further influenced by vascular-stromal-immune crosstalk. Endothelial cells and mesenchymal populations create metabolic and cytokine gradients that determine where T cells, B cells, and macrophages accumulate, polarize, and engage in effector functions [141]. This spatial heterogeneity underlies region-specific inflammation, immune cell clustering, and focal ECM remodeling, which collectively dictate the severity and architecture of fibrosis in MASH [142].

**Figure 4:**
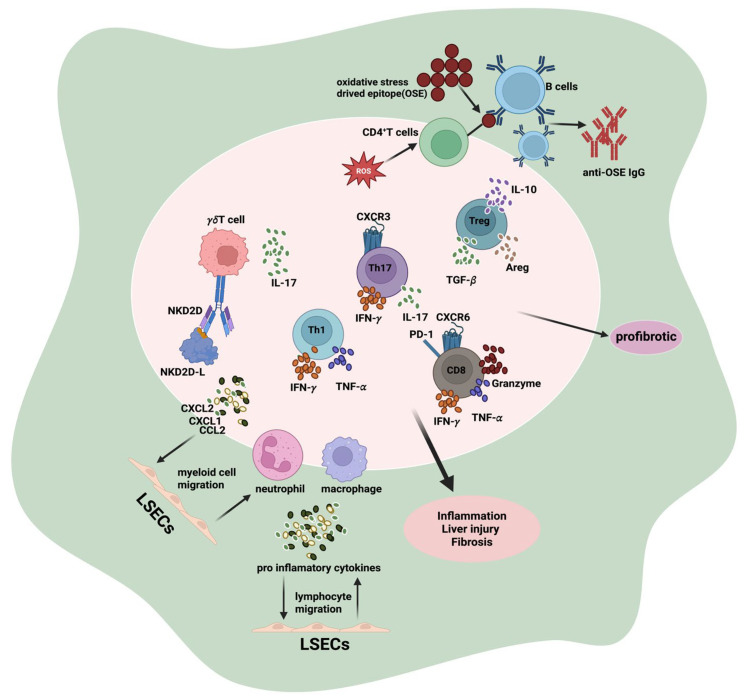
Adaptive immune mechanisms driving inflammation and fibrotic remodeling in MASH.

Finally, cross-talk between immune cells and heterogeneous stromal compartments contributes to dynamic feedback loops, where immune-derived signals prime stromal cells, which in turn reinforce immune activation and metabolic stress [143]. Such structured microenvironments emphasize that immune-stromal interactions are not uniform, but spatially organized networks that drive the progression from chronic inflammation to organized fibrosis, and ultimately influence clinical outcomes and therapeutic response (Figure 4).

Adaptive immunity critically contributes to MASH pathogenesis. Hepatocyte lipid overload induces oxidative stress, upregulating NKG2D ligands and activating γδ T cells to secrete IL-17, which stimulates hepatocytes to release CXCL1, CXCL2, and CCL2. These chemokines recruit macrophages and neutrophils, amplifying inflammation. Liver dendritic cells present oxidative stress-induced epitopes (OSEs) to CD4^+^ T and B cells, driving Th1/Th17 polarization and plasma cell differentiation with anti-OSE IgG production. Cytokines such as IFN-γ, TNF-α, and IL-17 exacerbate liver inflammation and fibrosis. Treg cells play a dual role, limiting fibrosis via IL-10 but promoting it through TGF-β and AREG. Overall, adaptive immune cells integrate metabolic stress with hepatic inflammation and fibrotic remodeling.

### 4.2. Chemokine-Mediated Recruitment and Spatial Remodeling

Chemokines are central mediators in shaping the spatial organization of immune responses within the liver during MASH. Specific chemokine families, such as CCL and CXCL members, establish gradients that direct the targeted recruitment of monocytes, macrophages, neutrophils, and lymphocytes to regions of active HSC engagement [144]. These localized accumulations of immune cells create hubs of concentrated cytokine signaling, allowing close interactions with stromal components and facilitating focused fibrogenic activity [145]. The heterogeneity of chemokine expression across hepatic tissue results in patchy, non-uniform fibrosis, reflecting the uneven distribution of recruited immune populations and activated stromal cells [146].

HSCs and endothelial cells express chemokine receptors, enabling them to sense and respond to immune-derived cues [147]. This bidirectional communication allows stromal cells to adjust their activation state, secrete additional chemokines, and sustain recruitment of immune populations [148]. Such feedback establishes microenvironments of heightened immune-stromal interaction, supporting ongoing ECM deposition and tissue remodeling [149]. Gradients of chemokine signaling act as both navigational cues and modulators of fibrotic patterning, integrating immune cell positioning with the spatial progression of fibrosis and forming localized niches of chronic inflammation and structural reorganization [150].

### 4.3. Feedback Loops Between Stromal Cells and Immune Cytokines

Fibrotic remodeling in MASH is driven not only by the linear effects of cytokines on stromal cells but also by dynamic feedback circuits that integrate multiple cellular players over time and space [151]. Within the liver, hepatic stellate cells and endothelial cells participate in these loops by sensing local cytokine environments and adjusting their secretory profiles, which in turn shapes the recruitment, activation, and retention of diverse immune populations [152]. This continuous interplay creates microanatomical niches where inflammatory and fibrogenic signals are locally amplified, generating persistent hotspots of tissue remodeling [153].

The complexity of these feedback loops lies in their multi-layered organization. Different immune cell subsets—ranging from monocytes and macrophages to T and B lymphocytes—contribute distinct cytokines that modulate stromal activation in complementary ways [154]. Stromal cells themselves secrete factors that influence immune cell differentiation, lifespan, and effector functions, effectively creating a bidirectional network in which the intensity, timing, and location of signals are tightly interdependent [155]. These interactions produce spatially heterogeneous fibrosis, with densely remodeled ECM regions coexisting alongside less affected areas, reflecting both the cumulative history of immune-stromal engagement and the local microenvironmental context [156,157].

Over time, such feedback loops can stabilize chronic inflammatory microenvironments, where the continued interplay between immune and stromal compartments reinforces tissue remodeling and promotes the patchy, organized patterns of fibrosis characteristic of MASH [158]. Conceptually, these observations highlight fibrosis as a dynamic, self-organizing system, in which structural remodeling emerges from reciprocal cellular communication rather than isolated signaling events [159].

### 4.4. Temporal Dynamics of Cytokine-Mediated Fibrotic Remodeling

Fibrotic remodeling in MASH represents a temporally orchestrated process in which immune-stromal interactions evolve over distinct phases [160]. The initiation phase is characterized by rapid recruitment of monocytes, macrophages, neutrophils, and T lymphocytes to areas of hepatocellular injury [161]. These cells release a surge of pro-fibrogenic cytokines, including TGF-β, IL-1β, TNF-α, and PDGF, which prime HSCs and endothelial cells for activation [162]. This early burst sets the stage for localized ECM deposition, establishing microenvironments that are predisposed to sustained remodeling [163].

As fibrosis progresses into the propagation phase, the cytokine milieu shifts. Regulatory and anti-inflammatory mediators, such as IL-10, CXCL9, and IL-22, emerge to modulate the intensity of HSC activation and ECM synthesis [164]. HSCs and endothelial cells dynamically adjust their secretory profiles in response to fluctuating cytokine concentrations, thereby shaping the recruitment and effector functions of incoming immune populations [143]. Feedback loops between stromal and immune compartments evolve simultaneously, either amplifying or dampening local inflammatory and fibrogenic activity depending on the cumulative signaling history [165].

During the maturation phase, spatially heterogeneous ECM networks solidify, with densely fibrotic foci forming in regions of persistent cytokine signaling while less exposed regions remain partially remodeled [166]. The timing, magnitude, and duration of cytokine signals determine the organization and density of fibrotic patches, explaining the characteristic irregular and patchy fibrosis observed in MASH histopathology [167]. Temporal fluctuations in chemokine gradients, stromal activation, and immune cell composition collectively drive the coordinated, yet heterogeneous, remodeling of liver tissue [168].

Taken together, the temporal dynamics of cytokine-mediated fibrosis underscore MASH as a continuously evolving system. Understanding these time-dependent processes highlights critical windows for therapeutic intervention, suggesting that modulating early pro-fibrogenic signals or interrupting sustained feedback loops could effectively limit the progression of chronic fibrotic remodeling [169].

## 5. Immune–Fibrotic Coupling as a Deterministic Driver of Clinical Outcomes

The coupling between immune activity and fibrotic remodeling exerts a deterministic influence on clinical outcomes, transcending mere correlation. Specific patterns of immune–fibrotic interaction—such as persistent macrophage infiltration coupled with dense ECM deposition, or high pro-fibrogenic cytokine levels in conjunction with activated hepatic stellate cells—can reliably predict the trajectory of disease progression from MASLD to MASH and ultimately advanced fibrosis [170]. Beyond progression, these immune–fibrotic signatures also inform therapeutic responsiveness: patients exhibiting a robust pro-fibrotic immune milieu may demonstrate reduced sensitivity to conventional anti-inflammatory or anti-fibrotic interventions, whereas modulation of these pathways can redirect remodeling trajectories and improve outcomes [171]. In this sense, immune–fibrotic coupling functions as a mechanistic determinant of disease course, providing both prognostic insight and a framework for personalized intervention.

### 5.1. Immune–Fibrotic Signatures and Clinical Severity

The clinical severity of MASLD and MASH is increasingly characterized through integrated immune–fibrotic signatures, which encompass immune cell distributions, cytokine networks, stromal activation markers, and ECM remodeling indicators [172]. While traditional studies have focused on individual molecular players, recent evidence demonstrates that the spatial organization and combined activity of these factors directly correlate with disease progression, fibrosis stage, and risk of hepatic decompensation [173].

Chemokines and cytokines play a central role in shaping both fibrotic microenvironments and clinical outcomes [174]. For example, chemokines such as CCL2, CCL5, CXCL10, and CXCL12 orchestrate monocyte and lymphocyte recruitment to fibrotic regions, creating dense immune microdomains that promote myofibroblast differentiation and ECM deposition [175]. Regulatory cytokines including IL-4, IL-13, and IL-22 modulate HSC activity and collagen organization, influencing fibrosis reversibility and structural integrity, which can determine progression from early-stage fibrosis to cirrhosis [176].

Peripheral blood immune profiling provides a minimally invasive window into disease severity [177]. Elevated levels of CCR2^+^ monocytes, CD14^+^CD16^+^ intermediate monocytes, skewed Th17/Treg ratios, and activated NK/NKT cell populations are consistently associated with higher fibrosis scores, increased hepatocyte injury, and greater likelihood of hepatic decompensation or MASH progression [178]. These peripheral signatures often mirror tissue-level immune heterogeneity, allowing early risk stratification before overt clinical deterioration occurs [179].

Hepatic tissue evaluation further refines the link between immune activity and fibrosis [180]. HSCs exhibit heterogeneous activation states, with distinct regions showing enhanced myofibroblast differentiation, fibronectin, laminin, and type V collagen deposition, which are associated with faster fibrotic progression and more severe clinical phenotypes [181]. Moreover, adaptive immune-derived cytokines, including IL-17, IL-21, IFN-γ, and lymphotoxin α/β, amplify HSC activation, leading to spatially heterogeneous fibrotic microenvironments [182]. These areas of high immune–fibrotic activity correspond to liver segments most prone to functional impairment and clinical complications [183].

Circulating biomarkers, such as soluble CD163, galectin-3, osteopontin, and matrix metalloproteinases (MMP-2, MMP-9), capture both ongoing inflammation and ECM remodeling [184]. These factors correlate with histological fibrosis stages and serve as predictive indicators for progression to advanced fibrosis or cirrhosis, offering additional clinical utility beyond standard biopsy or imaging [185].

Integration with non-invasive imaging techniques, including MR elastography, shear-wave ultrasound, and multiparametric MRI, allows quantitative and spatial mapping of immune–fibrotic activity [186]. By combining molecular, cellular, and imaging data, clinicians can identify regions at highest risk for progression, estimate fibrosis reversibility, and predict treatment response to targeted interventions [187].

Collectively, the integration of immune profiling, stromal markers, circulating biomarkers, and imaging enables the construction of composite scoring frameworks that link mechanistic insights to clinical outcomes [188]. These frameworks allow clinicians to stratify patients by risk, guide therapeutic decisions, and monitor disease trajectory dynamically, effectively translating molecular mechanisms into individualized clinical management.

### 5.2. Immune–Stromal Interactions in Disease Progression

The trajectory of MASLD and MASH is shaped by the dynamic organization of immune and stromal components, which manifests as both inter-patient variability and intrahepatic heterogeneity [189]. Patients with similar histological fibrosis stages can exhibit markedly different progression rates, highlighting the significance of the spatial and quantitative patterns of immune–stromal engagement [190]. Peripheral blood immune profiling, encompassing monocyte and lymphocyte subsets, NK and NKT cell activity, and regulatory T cell prevalence, offers early insight into these dynamics [191]. Alterations in these parameters often correlate with accelerated fibrotic progression, elevated risk of hepatic decompensation, and variable responses to therapy.

Integration of multi-parametric measurements, including chemokine gradients, soluble biomarkers such as galectin-3, osteopontin, and MMP-2, MMP-9, and imaging-derived assessments of tissue stiffness and fibrosis distribution, allows for a nuanced characterization of immune–stromal interactions [192]. These composite evaluations capture the heterogeneity of fibrotic remodeling, identifying focal zones of heightened immune infiltration and dense ECM deposition that are prone to rapid progression [193]. Non-invasive imaging techniques, such as MR elastography and shear-wave ultrasound, provide spatially resolved information that complements circulating biomarkers, revealing localized activity that may not be apparent in biopsy samples [194].

Clinically, mapping these immune–stromal landscapes enables refined patient stratification and individualized prognostication [195]. Patients exhibiting concentrated immune–stromal clusters or pronounced heterogeneity are more likely to experience rapid disease progression and may require closer monitoring or early therapeutic intervention [196]. Conversely, diffuse or moderate remodeling patterns are associated with slower progression, providing opportunities for preventive strategies [197]. Integrating these multi-dimensional data into predictive frameworks enhances risk assessment, informs treatment prioritization, and facilitates precision medicine approaches tailored to the patient’s unique immune–stromal profile [198].

### 5.3. Translational Insights and Future Directions

The integration of immune–fibrotic signatures into clinical practice holds substantial promise for advancing precision medicine in MASLD and MASH [199]. Multi-dimensional profiling—encompassing peripheral immune cell composition, circulating biomarkers, tissue remodeling indices, and non-invasive imaging—provides a comprehensive assessment of disease burden that surpasses conventional histological scoring [200]. These signatures not only reflect current disease severity but also enable stratification of patients based on predicted progression risk, likelihood of therapeutic response, and potential for disease regression [201].

Recent translational studies have demonstrated that incorporating immune–fibrotic metrics into clinical algorithms can improve prognostic accuracy [202]. For instance, composite scores integrating chemokine gradients, soluble biomarkers such as galectin-3 and osteopontin, and imaging-derived fibrosis patterns better predict progression to advanced fibrosis and cirrhosis than single-parameter models [203]. Moreover, the spatial heterogeneity of immune–fibrotic activity identified through imaging may guide targeted biopsy, optimize sampling strategies, and inform localized therapeutic interventions [204].

From a therapeutic perspective, immune–fibrotic profiling may inform patient selection for emerging interventions, including anti-inflammatory, anti-fibrotic, and immune-modulating agents [205]. Patients with concentrated immune–stromal clusters or highly heterogeneous remodeling patterns may require combination therapies or more aggressive early intervention, while those with diffuse, moderate remodeling may benefit from conservative or preventive approaches [206]. In addition, longitudinal monitoring of immune–fibrotic signatures offer a means to assess treatment efficacy dynamically and adjust therapeutic strategies in real time [207].

Future directions involve refining multi-omic integration, developing standardized scoring frameworks, and validating predictive models in large, diverse patient cohorts [208]. Advances in high-dimensional immune profiling, spatial transcriptomics, and functional imaging will likely reveal previously unrecognized patterns of immune–fibrotic interaction, enabling more precise patient stratification and the identification of novel therapeutic targets [209]. Ultimately, translating these insights into clinical workflows promises to improve patient outcomes, reduce progression to cirrhosis, and support individualized, mechanism-based management of MASLD and MASH [210].

## 6. Future Directions: Targeting Immunometabolic Circuits for Precision Therapy

Emerging evidence indicates that MASLD exhibits substantial heterogeneity in its immunometabolic profile across patients [211]. This variability not only influences disease progression but also impacts therapeutic responses, highlighting the need for precision medicine approaches [188]. Traditional “one-size-fits-all” interventions are often insufficient to address the complex interplay of metabolic, inflammatory, and immune pathways in individual patients [212]. Therefore, identifying patient-specific immunometabolic signatures is essential for designing targeted interventions that can effectively modulate disease trajectory, optimize clinical outcomes, and reduce the risk of complications [40]. This rationale underpins the necessity of precision therapy in MASLD and sets the stage for the following discussion on targeting immunometabolic circuits.

The preceding sections reveal that the major challenge in MASLD lies less in the complexity of individual mechanisms than in the diverse ways patients engage with them (Table 2). As precision medicine advances, focus is shifting from isolated pathways to understanding how a person’s immunometabolic profile shapes disease trajectory (Table 2). Future therapeutic progress will depend on integrating these individualized patterns to identify nodes capable of altering the course of disease [213].

With this perspective, the following sections will examine how immunometabolic signatures can guide patient stratification, highlight regulatory points with cross-stage therapeutic potential, and explore the relevance of multi-organ immunometabolic networks in developing individualized treatment strategies [214].

### 6.1. Integrating Immunometabolic Profiling into Clinical Stratification

In the context of precision medicine, leveraging immunometabolic information for patient stratification requires a framework that is both scientifically robust and directly applicable in clinical practice [215]. Achieving this involves integrating laboratory metrics, imaging features, and clinical measurements, while also accounting for dynamic changes and multi-dimensional correlations [216].

For example, MASLD-specific laboratory and imaging parameters, such as ALT/AST levels, MRI-PDFF liver fat fraction, liver stiffness measurements by FibroScan, and insulin resistance indices, can be combined to identify subgroups of patients at higher risk of rapid fibrosis or MASH development [217]. In addition, circulating inflammatory markers (e.g., IL-6, TNF-α) and metabolic biomarkers (e.g., CK-18 fragments, lipid intermediates) can be used to detect patients exhibiting early immunometabolic dysregulation [218].

For instance, modeling longitudinal follow-up data can reveal patterns linked to disease progression rates, likelihood of complications, and treatment responses [219]. Integrating these MASLD-specific biomarkers into predictive models allows clinicians to stratify patients into risk categories that reflect not only liver injury severity but also underlying metabolic and immune perturbations [220]. For instance, patients with elevated liver stiffness and persistent metabolic inflammation can be identified early, enabling consideration of lifestyle interventions, nutritional support, and enrollment in MASLD-focused clinical trials [221].

In practical terms, attention must be paid to data standardization, cross-center reproducibility, and model interpretability [222]. By training and validating predictive models on large cohort datasets, complex multidimensional information can be distilled into quantitative risk scores that assist clinicians in making intervention decisions [223]. This pattern-based stratification approach not only differentiates high- and low-risk patients but also allows dynamic adjustment of management strategies, enabling real-time optimization of treatment based on observed changes [224]. This MASLD-focused stratification provides a foundation for personalized monitoring schedules and early therapeutic planning, without yet specifying cross-stage or multi-organ interventions, which are discussed in Section 6.2 and Section 6.3.

This pattern-based stratification approach not only differentiates high- and low-risk patients but also allows dynamic adjustment of management strategies, enabling real-time optimization of treatment based on observed changes [225].When combined with lifestyle, metabolic status, and other clinical parameters, stratification outputs can more accurately guide intervention intensity and frequency, supporting individualized treatment and long-term management (Table 2) [226]. In this way, immunometabolic profiling becomes both a predictive and monitoring tool while providing a structured foundation for identifying actionable intervention points and multi-system integration [227].

### 6.2. Identifying Actionable Nodes for Cross-Stage Intervention

Within a precision medicine framework, the next critical step after patient stratification is to identify intervention nodes that exert meaningful influence across different stages of disease. These nodes are not defined by specific molecular or immune mechanisms but by their measurability, observability, and controllability within the clinical trajectory [228]. Analysis of longitudinal follow-up data, treatment responses, and multidimensional clinical metrics allows for recognition of nodes where early intervention can stabilize disease and nodes where mid-to-late adjustments can optimize long-term outcomes, enabling dynamic management from diagnosis through follow-up [229].

Implementation requires integration of diverse data sources into a continuous, actionable decision-making framework, allowing selection, sequencing, and intensity of interventions to be dynamically adjusted based on real-time observations [230]. Systematic monitoring can encompass traditional laboratory and imaging metrics as well as functional assessments and lifestyle data, creating a panoramic intervention strategy (Table 2) [230]. By incorporating overall patient characteristics, comorbid conditions, and individual preferences, interventions can be truly personalized and sustainable, ensuring that each decision aligns with long-term health goals [231].

Furthermore, this approach provides critical guidance for clinical research and novel therapy development. Identifying high-impact nodes and optimal intervention windows enables more precise clinical trial design, improving efficiency and interpretability of intervention strategies (Table 2) [232]. Cross-stage intervention frameworks also support long-term disease management, offering structured guidance to patients at different stages, while integrating stratification, dynamic intervention, and individualized decision-making into a cohesive model [233]. This strategy lays a solid foundation for future multi-system, multi-dimensional integrated therapeutic approaches.

### 6.3. Cross-System Integration and Multi-Organ Intervention

Within a precision medicine framework, single-organ interventions are often insufficient to address the systemic impact of MASLD and associated metabolic disorders [234]. Cross-system strategies emphasize positioning liver disease management within the context of the whole-body network, taking into account interactions between the liver and the cardiovascular system, kidneys, endocrine-metabolic pathways, gut microbiota, and neuroendocrine axes [235]. From this perspective, interventions extend beyond localized pathology to modulate key functional nodes across organs, enhancing overall therapeutic outcomes and reducing the risk of complications.

Implementing multi-organ intervention requires systematic integration of multidimensional data and decision support [236]. Clinically, non-invasive imaging, metabolic and laboratory markers, functional assessments, and longitudinal follow-up data can be used to construct models of inter-organ functional relationships [237]. These models reveal dynamic interactions between organs, providing evidence for clinical intervention (Table 2) [238]. For example, combined assessment of cardiovascular function and hepatic metabolic state can guide the prioritization of pharmacologic and lifestyle interventions, while monitoring renal and endocrine function helps optimize intervention intensity [239]. Integrating these multidimensional insights into actionable decision frameworks allows clinicians to adapt strategies dynamically across disease stages, achieving continuous, system-level management [240].

Cross-system interventions also provide a foundation for individualized therapy. Variability in organ function, disease burden, lifestyle factors, and comorbidities necessitates tailored intervention plans, ensuring sustainable and dynamically optimized treatment [241]. Multidisciplinary collaboration is essential: hepatology, cardiology, endocrinology, nephrology, and nutrition management can operate under a unified framework, coordinating interventions to complement each other, maximize efficacy, and minimize risk [242].

Furthermore, cross-system strategies inform clinical research and the development of novel therapies. Identification of critical nodes and sensitive intervention windows across organs enables more precise trial design, optimizes inclusion criteria and intervention combinations, and enhances interpretability and reliability of outcomes (Table 2) [243]. A structured framework for multi-organ intervention also supports long-term monitoring, providing continuous, coordinated guidance throughout disease progression [244]. This approach not only offers a comprehensive perspective for managing MASLD and its metabolic comorbidities but also lays the groundwork for future multidimensional, integrative therapeutic strategies.

**Table 2:** Comprehensive Cross-System and Multi-Organ Intervention Strategies (Compact & Comprehensive).

System/Organ	Intervention & Assessment	Integration Strategy & Stage	Potential Clinical Outcomes	References
**Liver**	FibroScan, MRI-PDFF, liver function, lipid metabolism; pharmacologic, diet, exercise, rehabilitation, nutritional interventions	Integrated with cardiovascular, renal, endocrine, and gut microbiota; early–mid stage	Improve fat accumulation, reduce inflammation, slow disease progression, enhance quality of life	[245]
**Cardiovascular**	Blood pressure, lipids, cardiac imaging, exercise tolerance; pharmacologic (antihypertensive, lipid-lowering), exercise, psychological, rehabilitation	Integrated with liver metabolism, renal function, neuro-endocrine axis; early–late stage	Reduce cardiovascular event risk, improve circulation, support long-term metabolic stability	[246,247]
**Kidney**	eGFR, proteinuria, fluid balance, electrolytes; pharmacologic, dietary management, fluid management, rehabilitation	Integrated with liver, cardiovascular, and endocrine parameters; mid–late stage	Protect renal function, reduce metabolic complications, maintain fluid-electrolyte balance	[248,249]
**Endocrine/Metabolic**	Blood glucose, insulin resistance, hormones, glycation markers; pharmacologic (glucose-lowering), nutrition, exercise, behavioral interventions	Integrated with liver lipid metabolism and cardiovascular function; early–mid stage	Improve insulin sensitivity, optimize metabolic status, reduce diabetes risk	[250,251]
**Gut Microbiota**	Microbiota diversity, SCFA, LPS levels, gut barrier; probiotics/prebiotics, dietary fiber, diet adjustment	Integrated with liver metabolism, immune status, and neuro-endocrine axis; all stages	Improve gut-liver axis function, reduce inflammation, optimize nutrient absorption	[252,253]
**Neuro-Endocrine Axis**	Hormones (cortisol, adrenaline), stress indicators, sleep quality; psychological, behavioral, cognitive interventions	Integrated with liver, cardiovascular, and metabolic parameters; all stages	Improve stress response, optimize systemic metabolism, promote lifestyle modification	[254]
**Multi-System Integrated**	Composite risk scores, dynamic monitoring, lifestyle, patient-reported outcomes (PRO), functional assessments	Cross-system integration for decision framework; across stages	Enhance overall disease control, reduce complications, optimize long-term management, improve quality of life	[255]
**Function & QoL**	Physical performance, exercise tolerance, daily activity, PRO; rehabilitation, behavioral, psychological interventions	Integrated with liver, cardiovascular, and metabolic indicators; mid–late stage	Improve quality of life, enhance self-management, mitigate functional decline	[256]
**Physiological Sensors & Dynamic Monitoring**	Continuous glucose monitoring, wearable cardiovascular/exercise sensors, sleep monitoring	Real-time feedback integrated with multi-organ indicators; all stages	Improve intervention precision, optimize dynamic management, support personalized decision-making	[257]

## 7. Pharmacological Interventions Targeting Immunometabolic Circuits in MASLD

Recent therapeutic strategies for MASLD focus on modulating immunometabolic circuits to simultaneously alleviate hepatocyte lipotoxicity, chronic inflammation, and fibrotic remodeling (Table 3). These pharmacological agents aim to restore metabolic homeostasis, suppress pro-inflammatory immune activation, and inhibit HSC fibrogenic responses. Main intervention categories include (i) lipid metabolism regulators, such as FXR agonists and ACC inhibitors, which reduce hepatic fat accumulation and downstream inflammatory signaling; (ii) cytokine/chemokine modulators, including IL-1β, TNF-α, and CCL2 inhibitors, which attenuate immune-stromal feedback loops; (iii) immune checkpoint modulators and Treg/Th17-targeting agents, which recalibrate adaptive immune responses and reduce hepatocyte cytotoxicity; and (iv) anti-fibrotic compounds targeting HSC activation through TGF-β, PDGF, or integrin signaling (Table 3). Several drugs exhibit multi-system effects, influencing not only liver pathology but also metabolic parameters, gut-liver axis signaling, and cardiovascular risk factors. Integrating these pharmacological approaches provides a framework for precision therapy in MASLD, emphasizing early intervention before irreversible fibrosis develops (Table 3).

## 8. Conclusions

MASLD represents a paradigmatic immunometabolic disorder in which hepatocellular stress, innate immune activation, and stromal remodeling converge to drive progressive steatohepatitis and fibrosis. Across Section 1, Section 2, Section 3, Section 4, Section 5 and Section 6 of this review, a coherent picture emerges: hepatocyte metabolic injury establishes an early immunologic threshold that dictates whether the liver maintains tolerance or transitions into chronic inflammation; innate and adaptive immune compartments integrate metabolic cues to shape disease trajectories; and immune–fibrotic coupling ultimately defines clinical severity, therapeutic responsiveness, and long-term outcomes.

A major conceptual advance highlighted in this review is that immune activation in MASLD is neither secondary nor epiphenomenal, but rather a central regulatory node governing tissue remodeling. Myeloid cells—including Kupffer cells, monocyte-derived macrophages, neutrophils, and dendritic cells—translate metabolic danger signals into coordinated inflammatory and fibrogenic programs. Concurrently, unconventional lymphocyte subsets, tissue-resident memory T cells, and metabolically reprogrammed effector populations contribute to a complex immune ecosystem that shapes spatial heterogeneity, temporal dynamics, and the persistence of disease. This multi-layered immune–stromal dialogue provides a mechanistic framework explaining why MASLD progression is highly variable among individuals despite shared metabolic risk factors.

Therapeutically, the field is moving beyond single-target strategies toward precision modulation of immunometabolic circuits. Interventions directed at metabolic checkpoints, immune cell bioenergetics, cytokine–stromal signaling axes, and gut–liver crosstalk hold promise for interrupting maladaptive immune activation while preserving essential tissue-protective functions. Integrative approaches—combining systemic metabolic correction, organ-specific immunomodulation, and restoration of tissue homeostasis—represent the next frontier. Moreover, multi-omics profiling, spatial single-cell technologies, and computational modeling are poised to refine patient stratification and prediction of therapeutic response, accelerating the translation of mechanistic insights into personalized management.

In summary, MASLD progression is fundamentally an immunometabolic process driven by sustained interactions among hepatocytes, immune cells, and stromal compartments. Continued integration of basic mechanistic research with biomarker development and targeted intervention will be essential to transforming current management and achieving true precision therapy for MASLD.

## Figures and Tables

**Table 1:** Key Immunological Indicators Reflecting Immune Cell Reprogramming in MASLD.

Immune Cell Type	Dysfunctional Features	Key Molecular/Signaling Markers	References
Kupffer Cells (KCs)	Shift toward pro-inflammatory phenotype; impaired phagocytosis	Clec4f ↓, Tim4 ↓; TNF-α ↑, IL-1β ↑; NLRP3 activation	[118,119]
Monocyte-derived Macrophages (MoMFs)	Massive infiltration; amplified inflammatory response	CCR2/CCR5 ↑, CX3CR1 ↑; Ccl2 ↑; S100A8/A9 ↑	[120,121]
Dendritic Cells (DCs)	Enhanced antigen presentation or tolerogenic skewing	CD80/CD86 ↑; CD103+ DC linked with Treg induction	[122,123]
Neutrophils	Increased NET formation; promote hepatocyte injury	MPO ↑, NE ↑; PAD4 ↑	[124]
NK Cells	Altered cytotoxicity; dual roles in fibrosis	NKG2D shifts; IFN-γ fluctuations; TRAIL signaling	[125,126]
NKT Cells	Reduced number but pro-inflammatory shift	CD1d ↓; altered IL-4/IFN-γ ratio	[127]
γδ T Cells	Contribute to early inflammation and impaired regeneration	IL-17A ↑; RORγt ↑	[128]
CD4^+^ T Cells	Th1/Th17 polarization; Treg dysfunction	IL-17A ↑, IFN-γ ↑; Foxp3 ↓, IL-10 ↓	[129,130]
CD8^+^ T Cells	Enhanced cytotoxicity; drive hepatocyte killing	GzmB ↑, Perforin ↑; CXCR6 ↑	[131]
B Cells	Autoantibody production; expanded pro-inflammatory B2 cells	BAFF ↑; IgA ↑; CD19+ B2 cell expansion	[132,133]

↑ indicates an increase; ↓ indicates a decrease.

**Table 3:** Therapeutic Agents Modulating Immunometabolic and Fibrotic Pathways in MASLD.

Drug/Compound	Primary Target	Mechanism of Action	Multi-Organ/Immunometabolic Effects	Clinical Stage	References
**Obeticholic acid**	FXR	Reduces hepatic lipogenesis, enhances bile acid signaling, suppresses pro-inflammatory cytokines	Improves insulin sensitivity, modulates gut-liver axis	Phase III	[258]
**Selonsertib**	ASK1	Inhibits stress-activated JNK/p38 signaling, reducing HSC activation	Attenuates oxidative stress and apoptosis	Phase III	[259]
**Cenicriviroc**	CCR2/CCR5	Blocks monocyte/macrophage recruitment, limits HSC activation	Modulates immune-stromal feedback, reduces systemic inflammation	Phase III	[260]
**Resmetirom**	THR-β	Enhances hepatic lipid oxidation, decreases steatosis	Improves metabolic profile, reduces pro-inflammatory signals	Phase III	[261]
**Galectin-3 inhibitors**	Galectin-3	Inhibits ECM deposition, reduces HSC activation	Modulates immune cell recruitment and fibrosis	Phase II	[262]
**IL-1β inhibitors**	IL-1β	Suppresses pro-inflammatory signaling	Reduces liver inflammation and systemic metabolic dysfunction	Phase II	[263]
**FGF19 analogs**	FGF19 receptor	Regulates bile acid metabolism and glucose homeostasis	Reduces lipotoxicity, modulates immune cell activation	Phase II	[264]
**FGF21 analogs**	FGF21 receptor	Improves lipid and glucose metabolism	Reduces hepatocyte stress, modulates macrophage polarization, enhances Treg activity	Phase II	[265]
**Thyroid hormone receptor-β agonists**	THR-β	Enhances fatty acid oxidation, reduces hepatic lipid accumulation	Decreases hepatic inflammation, indirectly modulates innate and adaptive immune responses	Phase I/II	[266]
**Gut-restricted FXR/FGF19 modulators**	FXR/FGF19	Localized modulation of bile acid signaling and microbiota-liver crosstalk	Improves gut barrier, reduces portal endotoxin exposure, modulates Kupffer cell activation	Early clinical	[267]

## Data Availability

No new data were created or analyzed in this study. Data sharing is not applicable to this article.
